# Spatial–temporal distribution of *Aedes* (*Stegomyia*) *aegypti* and locations of recycling units in southeastern Brazil

**DOI:** 10.1186/s13071-019-3794-z

**Published:** 2019-11-14

**Authors:** Rafael Piovezan, Alexandre Visockas, Thiago Salomão de Azevedo, Cláudio José Von Zuben, Maria Anice Mureb Sallum

**Affiliations:** 1 Departamento de Zoologia, Universidade Estadual Paulista, Rio Claro, SP Brazil; 2 Department of the Environment, Prefeitura Municipal de Santa Bárbara d’Oeste, Santa Bárbara dʼOeste, SP Brazil; 3 Departamento de Epidemiologia, Faculdade de Saúde Pública, Universidade de São Paulo, São Paulo, SP Brazil

**Keywords:** *Aedes aegypti*, Vector control, Material recycling, Dengue, Spatial distribution

## Abstract

**Background:**

Dengue is an arbovirus disease that threatens approximately 200 million people annually worldwide. *Aedes* (*Stegomyia*) *aegypti* (Linnaeus, 1762) is anthropophilic mosquito, extremely well adapted to the urban environment and utilizes varied habitats for egg-laying and development. This study analysed the distribution of mosquito larvae and eggs in urban area of Santa Bárbara dʼOeste, São Paulo, Brazil. The spatial correlation between locations in which people store recyclable materials and the distribution of larvae and eggs were verified.

**Methods:**

Larvae and ovitrap egg collections were conducted from 2014 to 2016. All persons who stored recyclable materials for living were registered and georeferenced. The Mann-Kendall test was used to verify spatial and temporal trends in the number of eggs and larvae/pupae. Euclidian distance map was constructed to correlate recyclable collectors and *Ae. aegypti*, and Moranʼs index was employed to verify their spatial autocorrelation and identification of groupings.

**Results:**

A total of 137,825 eggs and 16,393 larvae were collected in different habitats from 2014 to 2016. The analyses showed that there was a spatial correlation between larvae and eggs collected, and these two kinds of surveys also presented a spatial correlation with the handling of recyclable materials. The results of the analyses showed significant spatial correlations between eggs and recyclable material collectors and between larvae and collectors.

**Conclusion:**

The entomological surveillance conducted using ovitraps as a proxy for the presence of *Ae. aegypti* is an efficient and sensitive method for monitoring the presence of mosquitoes and the impact of interventions employed for decreasing vector populations. Mosquito surveys employing ovitraps should be used more often in routine activities aiming to control dengue through vector control interventions. The locations used to store recyclable materials have a significant relationship with the maintenance of the dengue virus infection in the area. Further studies will be needed to analyse the contribution of recyclable locations, for which there is no ideal infrastructure to minimize the potential use of these materials as mosquito habitats. The entomological surveillance focused on locations of recyclable materials involving interventions that are different from those commonly used in *Ae. aegypti* control.
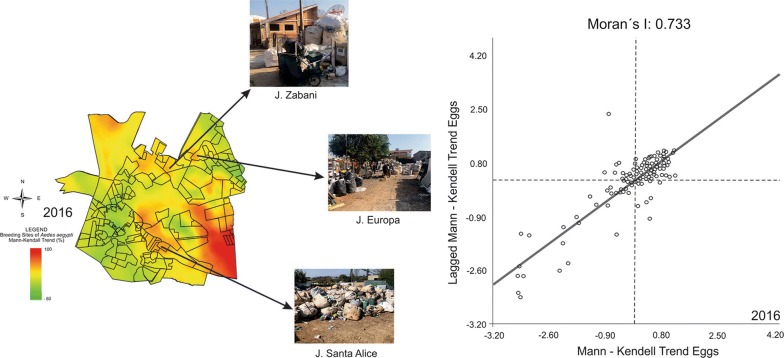

## Background

Mosquitoes (Culicidae) are of great epidemiological importance because of their role in the transmission of several pathogens [1]. These insects are estimated to threaten 2.5 billion people worldwide in terms of dengue virus infections [2]. In addition, mosquito species are vectors of other globally important pathogens, such as those that cause malaria, filariasis, chikungunya, Zika fever and yellow fever [3, 4].

*Aedes* (*Stegomyia*) *aegypti* (Linnaeus, 1762) is recognized as the most important vector species of human pathogens in urban areas due to its ability to adapt to multiple larval habitats [5, 6]. The vast majority of larval habitats are man-made [1, 7, 8], and this characteristic is the greatest challenge of *Ae. aegypti* vector control programmes. Another important issue that exacerbates the situation is the absence of efficient administration of solid waste disposable materials that can become potential habitats for *Ae. aegypti* [9]. *Aedes* (*Stegomyia*) *albopictus* (Skuse, 1894) is an opportunistic outdoors, day biter mosquito, inhabiting a wide range of habitats, such as vegetated rural and peri-urban areas in Brazil. This species is competent to transmit several arboviruses, such as the yellow fever virus [4].

The success of *Ae. aegypti* control is influenced by the interventions employed, such as active larval and pupae surveys with their subsequent removal, and adult control with dawn or dusk ultra-low-volume (ULV) insecticide applications and monitoring for resistance. In the urban environment, the impact is measured by various indices of mosquito infestation [10, 11]. Breteau’s index, the predial index and the recipient index [12] are largely employed by *Ae. aegypti* control programmes. They are metrics that involve home inspections with random sampling. Breteau’s index is the proportion between the number of properties surveyed for mosquitoes and the number of positive habitats found. The impact of a control programme focused on the control of *Ae. aegypti* depends on the employment of effective actions to eliminate the majority of potential habitats to reduce human exposure to mosquito bites [6].

Dengue is among the most globally important vector-borne diseases caused by RNA viruses of the genus *Flavivirus*. Five dengue serotypes (DEN1, DEN2, DEN3, DEN4 and DEN5) have been identified [13, 14]. Infection can cause symptoms that include high fever, myalgias, arthralgia, headache and in severe cases, dengue shock syndrome and haemorrhagic dengue fever [13, 14]. Globally, the average number of reported dengue cases reached approximately 9387 million in the last six years [15]. However, these reported cases, confirmed by laboratory tests, underestimate the impact of dengue infection because the estimates of annual infections are frequently orders of magnitude higher (390 million infections per year) [16–19]. Most dengue virus infections are asymptomatic or mild (only one in four patients is symptomatic), but approximately 5% of infected people may develop severe dengue. The epidemiological importance of asymptomatic individuals suggests that this condition becomes a complicating factor in disease control, representing a silent reservoir of the virus that continues to operate and carry out all usual activities [20].

In Brazil, dengue viruses are primarily transmitted by *Ae. aegypti* [21]. In 2015 and 2016, Brazil reported 1.688 and 1.483 million cases, respectively [22]. In 2017 and 2018, dengue incidences decreased, but the number of cases (239,389 in 2017 and 265,934 in 2018) was worrisome, demanding the attention of control programmes [23]. In the four years noted, approximately 2000 deaths resulting from dengue were registered, and more than 3000 serious cases of dengue were also reported [22, 23].

Beyond the threat posed by dengue, Brazil is impacted by other emerging arboviruses whose aetiological agents are also transmitted by *Ae. aegypti*. These include chikungunya fever, with 277,882 cases reported in Brazil in 2016, 185,593 in 2017 and 87,687 in 2018, whereas the number of Zika fever cases was 216,207 in 2016, 17,593 in 2017 and 8680 in 2018 [23]. The aggravating factors of Zika virus infection are the alterations it produces in the development and growth of foetuses and children. Since epidemiological week 45 in 2015, there have been 3279 confirmed cases with adverse outcomes, including congenital Zika syndrome and microcephaly, in Brazil [24].

The interventions that focus on the control of *Ae. aegypti* encompass the surveillance and control of the mosquito, epidemiological surveillance of both suspected and confirmed cases of the disease, and social communication and health education [10, 11]. According to the Brazilian Ministry of Health, the annual financial investment in vector control programmes focusing on *Ae. aegypti* in Brazil has to date exceeded USD$ 300 million [25].

Entomological surveillance and control of *Ae. aegypti* present great challenges for public health. The search for methods that would help optimize entomological control permits, beyond financial support given to programmes, are directed to the points of greatest environmental and epidemiological risk, which receive preferential treatment [17, 26–28]. Therefore, effective control of *Ae. aegypti* results in the reduction of the risk of acquiring dengue infection and other arboviruses [1, 29]. Thus, the use of oviposition traps in surveillance programmes focused on *Ae. aegypti* is a sensitive and safe way of helping coordinate mosquito control [30, 31]. Within this context, validation of the methods usually employed and available for the control of *Ae. aegypti* is fundamental to guaranteeing the effectiveness of the measures used for the control of this vector and for the safety of society with regard to the various arboviruses linked to this vector [4]. Thus, our study aimed to (i) evaluate the implementation of ovitraps as an additional method for entomological surveillance in the geographical region of the study, and (ii) determine the influence of the spatial distribution of locations used to store recyclable materials on the occurrence of *Ae. aegypti* larval habitats.

## Methods

### Study area

**Fig. 1:**
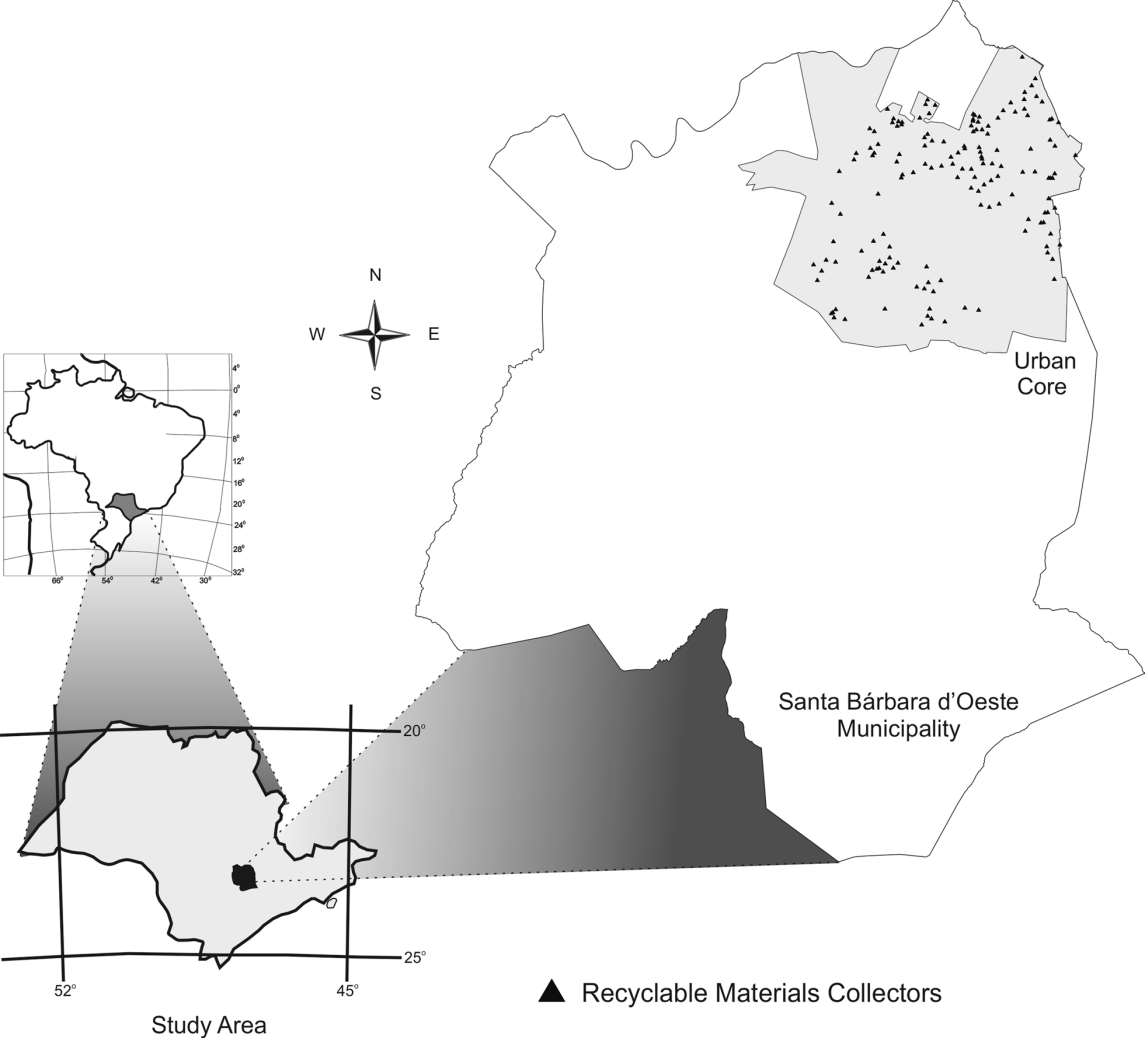
Location of the study areas in Santa Bárbara dʼOeste, São Paulo State, Brazil

The present study was conducted in the municipality of Santa Bárbara d’Oeste, São Paulo State, southeastern Brazil (Fig. 1) (22°45′17″S, 47°24′52″), at an average altitude of 560 m. The total area of the municipality is approximately 271 km^2^. Based on the classification proposed by Koeppen [32], Santa Bárbara d’Oeste possesses a climate defined as Cwa, characterized as high-level tropical, with summer rains and dry winters. The average temperatures of the hottest and coldest months are above 22 °C and 12 °C, respectively. The average annual rainfall is 1,466.1 mm [33, 34].

### Field collection

**Fig. 2:**
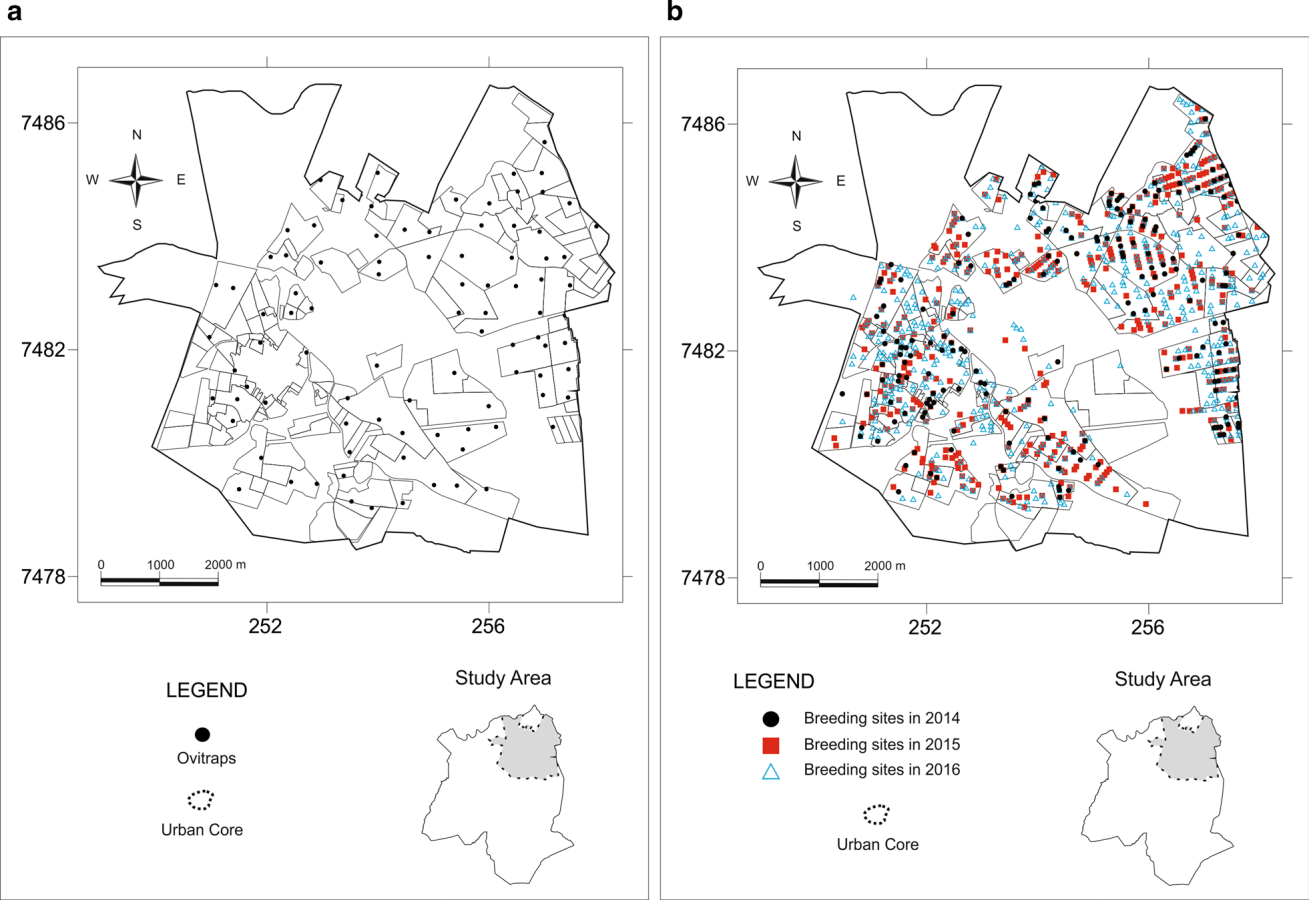
Location of the ovitraps (**a**) and breeding sites (**b**), Santa Bárbara dʼOeste, São Paulo State, Brazil

Two methods were employed for the mosquito collection, i.e. larvae were taken from different kinds of habitats daily during a house-to-house survey. Only positive habitats were recorded. For egg surveys, 155 mosquito ovitraps were placed throughout the municipality. Both methods are described in manuals for dengue control [10, 11, 21]. During the three-year study, interventions for vector control were carried out in various areas of the municipality by the same group of technical personnel using the same operational and technical skills. Field collection occurred from 2014 to 2016 (Fig. 2). Larval habitats were eliminated after larval/pupal collections, and the residents were taught about the procedures to avoid the emergence of new mosquito habitats both indoors and outdoors. This is part of an educational programme that is conducted together with *Aedes* control. Houses were monitored every two months throughout the year.

### Collection of larvae

Larvae collections were undertaken by the mosquito personnel of the Municipal Health Secretariat. The personnel surveyed human dwellings in the urban area of the municipality from 2014 until 2016, collecting larvae and pupae from all habitats they found at each location. During the entomological survey, mosquito larvae were collected. Immature larvae collected were separated, and the kinds of habitats were identified and annotated. Later, all the materials with the larvae collected were identified, the larvae of each on them were counted, and in the laboratory, identification of the specimens was undertaken using the morphological key by Forattini [1]. This information was organized and geo-referenced.

### Ovitraps

One hundred fifty-five ovitraps were distributed in the urban area of the municipality and surveyed every week each year (2014, 2015 and 2016). They consisted of a black plant pot with an approximate volume of one litre of fresh water and a wooden pallet 14 cm long, 3.5 cm wide and 0.5 cm thick. The distribution of the traps was determined by overlaying a grid with squares on the map of the municipality, considering the information on the spatial correlation obtained by Piovezan et al. [8], that is, establishing a distance of a radius of approximately 270 m between the traps.

The ovitraps were installed in the peridomicile of the houses, approximately 1.5 m above ground level. The traps were inspected by mosquito control personnel, and the pallets were removed from the ovitraps and immediately replaced by new ones of the same dimensions, material and characteristics, but without any eggs. The water in the recipient material was replenished, and a new collection was carried out in the following week. Pallets were stored in the laboratory to dry, and later, the eggs on each substrate were counted under a stereomicroscope; the numbers were recorded in a data set. The eggs were systematically removed from the pallets by means of brushes and disposed of in the sewage network. Thus, the disposed eggs were taken to a point at a distance from the urban area of the municipality, with their destination being River Piracicaba. The egg collected *via* the ovitraps involved standardized methods routinely employed for monitoring *Ae. aegypti* and *Ae. albopictus*. As with the data on the larvae, these data were also recorded in a data set and geo-referenced.

### Recyclers survey

The survey of the recyclers was undertaken by the endemic control agents with the help of the Municipal Environmental Secretariat (Secretaria Municipal de Meio Ambiente). All persons who stored recyclable materials for a living were registered and georeferenced. Notably, the recyclers referred to here are those persons who engage in the active collection of recyclable materials in residences. They are responsible for great volumes of collected materials, storing these materials in fixed locations, which are frequently their own residences. Previous sorting can be observed in these places during which time the materials were separated by type and later commercialized and transported to larger deposit areas or even directly to firms that specialize in recycling activity. Recyclable materials comprised several kinds of manufactured goods made of glass, metal and plastic.

### Spatial analysis

To determine the spatial and temporal trends in the number of eggs and larvae/pupae, the Mann-Kendall test was used [35, 36]. This test is a robust, sequential and non-parametric test used to analyse a series of data. An advantage of this test is that it is only minimally influenced by abrupt changes or by a non-homogeneous series [37]. The variable statistic S for a series of data N of the Mann-Kendall test was calculated based on time (t) with regard to the values in t + 1 (xj), expressed in equations (1) and (2).

1 $${\text{S}} = \mathop \sum \limits_{{{\text{i}} = 1}}^{{{\text{n}} - 1}} \mathop \sum \limits_{{{\text{j}} = {\text{i}} + 1}}^{\text{n}} {\text{sgn}}\left( {{\text{x}}_{\text{j}} - {\text{x}}_{\text{i}} } \right)$$

2 $${\text{sgn}}\left( {{\text{x}}_{\text{j}} - {\text{x}}_{\text{i}} } \right) = \left\{ {\begin{array}{*{20}c} { + 1;} & {{\text {se}} \;{\text{x}}_{\text{j}} > {\text{x}}_{\text{i}} } \\ {0;} & {{\text {se}} \;{\text{x}}_{\text{j}} = {\text{x}}_{\text{i}} } \\ { - 1;} & {{\text {se}} \;{\text{x}}_{\text{j}} < {\text{x}}_{\text{i}} } \\ \end{array} } \right.$$

This method, however, requires that the data be separated and random; thus, the Mann-Kendall contextual (CMK) test was applied [38]. This calculation was adopted because geographically neighbouring regions tend to have similar characteristics. The Mann-Kendall test was carried out individually on each pixel, without considering the behaviour of the neighbouring pixels. The CMK method is non-parametric and consists of the regionalization of the series [39]. The magnitude of the autocorrelation must be considered in the test of significance. To undertake this analysis, a mask of the average of the pixels with the dimension 2 × 2 after the calculation of the variable S of the Mann-Kendall test was applied.

The data were compiled and analysed using the Idrisi Selva 17.02 GIS. The Earth Trends Modeler module was used to analyse tendencies in the spatially distributed historical series. The result of this analysis was a compilation of maps showing the spatial tendency of the eggs and the larvae, thus determining which regions had undergone changes over time.

To ascertain the behaviour of the Euclidian distance, a map of the collectors of recyclable materials was compiled. This procedure was undertaken in ArcGis10.2. The map of Euclidian distances measured the distance in a straight line from each recycler to the nearest recycler. After the compilation of this chart, both the data set of the larval habitats of *Ae. aegypti* and the eggs were overlaid. A spatial analysis tool to determine the distances of these entomological parameters in relation to the collectors of recyclable materials was employed. Histograms were then developed to show the behaviour of the larval habitats, presence of eggs in ovitraps and recyclable locations graphically.

To correlate the collectors of recyclable materials and the eggs and larvae of *Ae. aegypti*, a local multivariate indicator of the spatial association (multivariate local indicator of spatial association, LISA) was employed. This statistical method allowed us to assess the hypothesis of spatial randomness by comparing the values of the indicator of each region with the indicators of neighbouring regions. The Moranʼs local I (Ii) carries out the spatial autocorrelation of a specific locality with its neighbours, permitting the identification of groupings [40].

This index was used because according to [41], when one is working with a large number of areas, it is possible that different regimes of spatial association may occur. Thus, the use of multivariate global indicators of social correlation might underestimate spatial correlations because only an average value of spatial association is calculated for the whole collection of data.

## Results

### Ovitraps

**Fig. 3:**
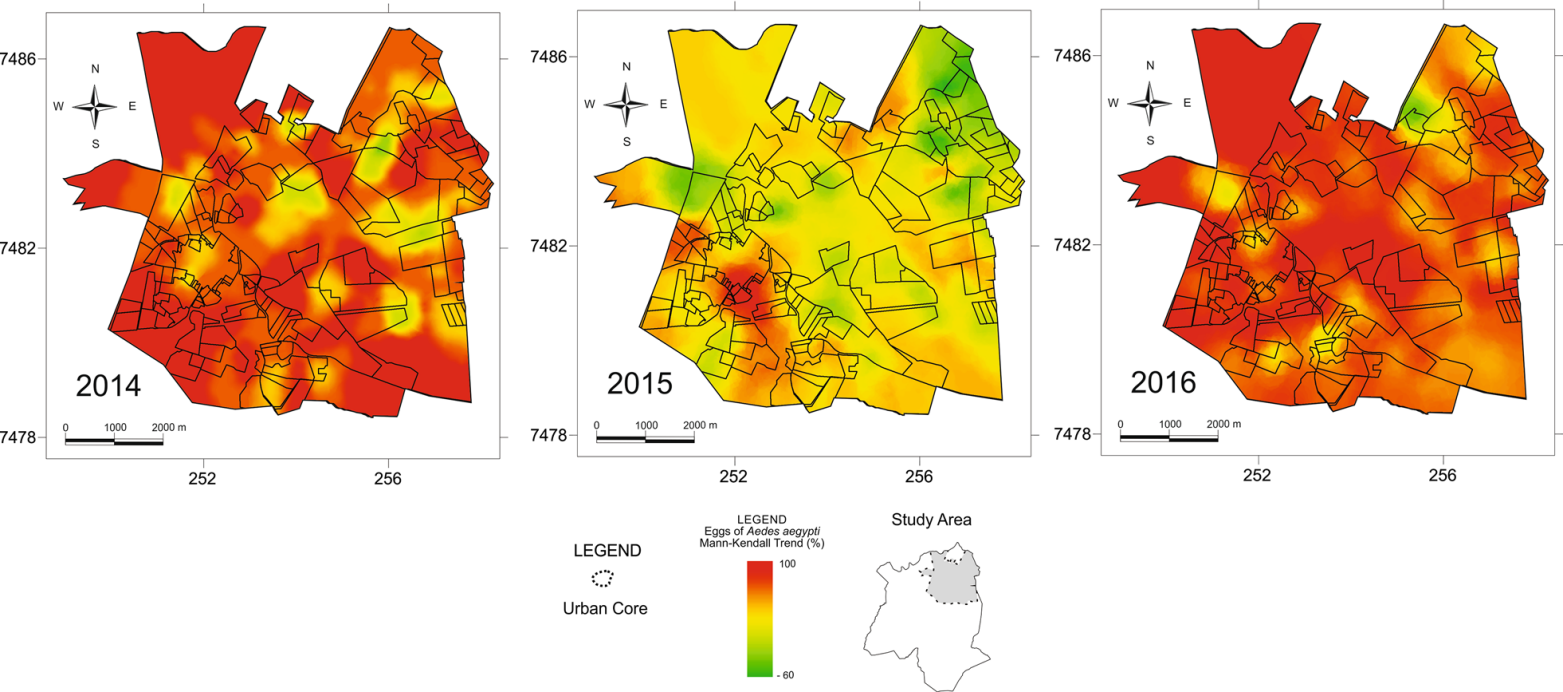
Mann-Kendall analysis of the distribution of eggs collected in the ovitraps (2014, 2015 and 2016)

**Fig. 4:**
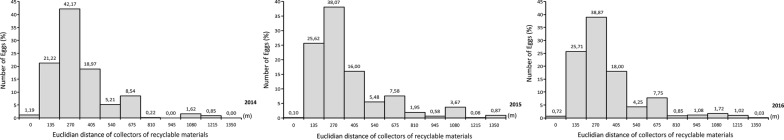
Number of eggs collected in the ovitraps *versus* the distance from the collectors of recyclable materials

During the ovitrap survey, 137,825 eggs were taken from the traps. The average number of eggs collected weekly from each of the traps was 19.87 eggs (2014), 7.76 eggs (2015), and 15.3 eggs (2016). The number of eggs found in 35 traps were above the average in 2014; 49 traps in 2015; and 51 traps in 2016. The results of the Mann-Kendall analysis focusing on mapping the eggs collected in the traps demonstrated a heterogeneous distribution of the eggs, presenting distinct values for each region of the municipal territory in each year of collection (Fig. 3). The distribution of the number of eggs collected in the traps by the Euclidean distance of the recyclers demonstrated that 64.58% of the eggs that were collected in the traps were at a distance of up to 270 m from the recyclers. In 2015, 63.79% of the total number of eggs and in 2016, 65.3% of the total number of eggs were collected in traps at a distance of up to 270 m from the recyclers (Fig. 4).

### Collection of larvae

**Fig. 5:**
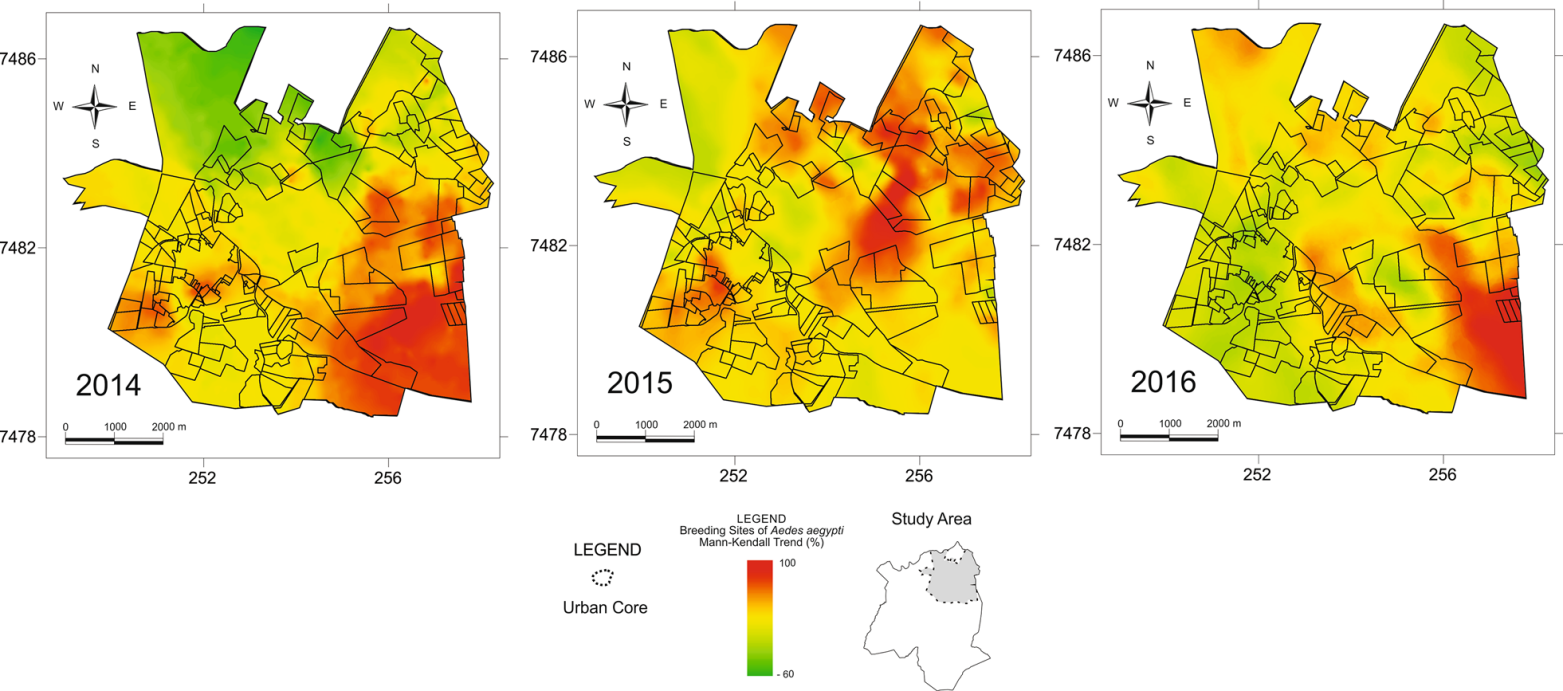
Mann-Kendall analysis of the distribution of larvae collected at different breeding sites (2014, 2015 and 2016)

**Fig. 6:**
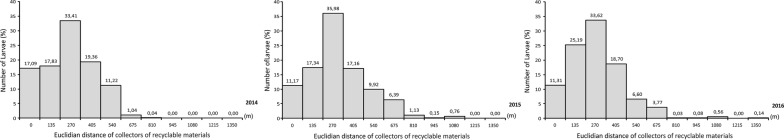
Number of larvae collected at different breeding sites *versus* their distances from the collectors of recyclable materials

The surface maps of the trend in the distribution of larvae in the municipality territory demonstrate the different concentrations in each area surveyed (Fig. 5). A total of 16,393 larvae of *Ae. aegypti* were collected in different types of breeding habitat during 2014, 2015 and 2016. The distribution of the larvae in each type of habitat is shown in Table 1. Similar to the distribution of the positive ovitraps, the results of the analysis focusing on the larval distribution revealed that the majority of the habitats where specimens were found are located at distances of less than 270 m from the recyclers (Fig. 6).

**Table 1:** Distribution of the larvae collected in different kinds of habitats in Santa Barbara D’Oeste, State of São Paulo, Brazil, from 2014 to 2016

Breeding site	Kind of breeding site	2014		2015		2016		General	
		Larval habitat (%)^a^	Larva (%)^b^	Larval habitat (%)^a^	Larva (%)^b^	Larval habitat (%)^a^	Larva (%)^b^	Larval habitat (%)^c^	Larva (%)^d^
Raised deposit	1. Raised deposit connected to electrical grid	2.05	1.19	0.27	0.21	0.79	0.21	0.76	0.35
	2. Raised deposit un-connected to the grid	0	0	1.79	1.38	2.86	1.85	2.11	1.40
Unraised deposit	3. Unraised deposit connected to the grid	0.41	0.08	1.24	0.77	0.20	0.14	0.60	0.37
	4. Unraised deposit un-connected to the grid	4.10	10.38	13.05	8.95	12.43	10.06	11.63	9.69
Furniture	5. Vase with plant in the water	9.43	11.16	3.57	2.17	1.68	1.64	3.32	3.26
	6. Various vases with plants	4.51	2.26	3.71	4.24	5.13	6.32	4.53	4.93
	7. Plate with plant/water dispenser	17.62	11.89	6.46	7.12	7.20	6.46	8.21	7.52
	8. Animal consumption	2.05	1.44	2.34	2.41	2.47	2.57	2.37	2.34
	9. Store for construction	0.41	0.04	0	0	0.10	0.01	0.10	0.01
	10. Shed for horticulture	0	0	0.14	0.02	0.20	0.13	0.15	0.07
	11. Portable swimming pool	1.64	0.49	0.55	1.08	1.18	1.11	1.01	1.01
	12. Metal can, flask, used plastic	9.02	15.91	5.77	5.92	3.94	3.25	5.24	6.14
	13. Returnable bottles	0	0	1.65	1.43	0.39	0.12	0.81	0.60
	14. Bucket, watering can	7.38	5.58	7.97	6.98	10.55	8.07	9.21	7.28
	15. Tray of refrigerator/air conditioning	0.41	0.12	0.27	0.08	0.30	0.35	0.30	0.21
	16. Building material	0.41	0.04	0.14	0.06	0	0	0.10	0.03
	17. Other movable breeding sites	2.05	1.07	5.08	3.25	2.86	3.21	3.58	2.90
Fixed	18. Internal grating	0	0	0.14	0.03	0.39	0.28	0.25	0.15
	19. External grating	2.87	0.70	2.47	1.91	3.35	3.89	2.97	2.67
	20. Concrete slab	0	0	0	0	0	0	0	0
	21. Gutters	0	0	0.69	0.92	0.99	0.58	0.76	0.62
	22. Toilet basin/flush tank	4.10	4.76	3.85	3.55	2.76	2.19	3.32	3.09
	23. Swimming pool	0.41	0.04	0.55	0.82	0.39	0.48	0.45	0.54
	24. Shed for building materials	0	0	0	0	0.20	0.12	0.10	0.05
	25. Shed for horticulture	0	0	0.14	0.05	0.10	0.17	0.10	0.10
	26. Animal food plate	0.41	0.16	1.10	0.95	0.30	0.48	0.60	0.61
	27. Other fixed breeding sites	0.82	0.49	0.82	0.79	1.38	0.72	1.11	0.71
Tires	28. Tire	4.51	5.82	4.53	7.70	6.21	7.53	5.39	7.34
	29. Others related to tires	0	0	0	0	0.10	0.12	0.05	0.05
Subject to removal/alteration	30. Metal can, plastic flask	6.97	14.19	7.14	12.55	9.37	14.03	8.26	13.49
	31. Returnable bottles	0.82	0.78	0.41	0.55	0.49	0.14	0.50	0.39
	32. Canvas. tarpaulin, plastic sheet	3.28	2.26	5.91	7.10	5.42	6,76	5.34	6.22
	33. Rubble from construction	0	0	0.27	0.19	0.20	0.08	0.20	0.11
	34. Parts/scrap	1.23	1.64	1.37	0.96	2.86	4.86	2.11	2.90
	35. Plaster recipient	0	0	0.27	0.10	0.10	0.09	0.15	0.08
	36. Boat	0	0	0	0	0.10	0.01	0.05	0.01
	37. Other removable items	10.25	6.32	13.19	13.71	9.86	9.50	11.13	10.63
Natural	38. Tree trunk	0	0	0.27	0.26	0.10	0.01	0.15	0.10
	39. Bromeliads	2.87	1.19	1.24	0.42	0.99	0.62	1.31	0.63
	40. Other natural habitats	0	0	1.65	1.37	2.07	1.84	1.66	1.38

^a^Percentage of different recipients used as habitats for immature stages in relation to total number of habitats searched and found positive for larvae in a specific year

^b^Percentage of larvae collected in each group of recipients in relation to the total number of larvae collected in a specific year

^c^Percentage of different habitats for immature stages in relation to total number habitats searched and found positive for larvae from 2014 to 2016

^d^Percentage of larvae collected in different habitats in relation to the total number of habitats searched and found positive for larvae from 2014 to 2016

### Recycling sites

**Fig. 7:**
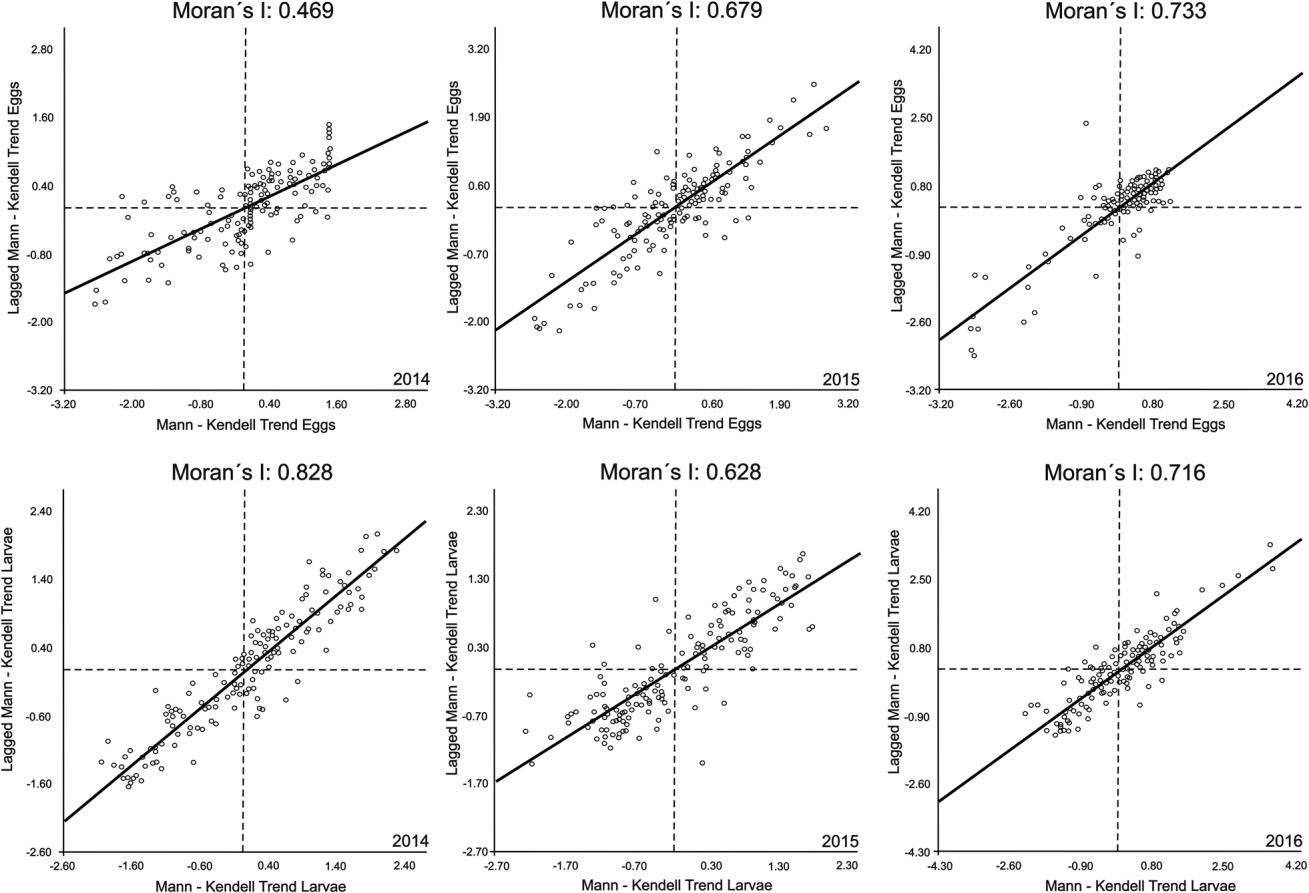
Moran’s index: correlation between the space-time trend in larvae and eggs collected and the distribution of recyclers

The results of Moran’s index correlated the presence of the recyclers to the spatial–temporal Mann-Kendall trend, demonstrating that there was a positive correlation between the spatial–temporal trend in larvae and the distribution of recyclers. The results of the Moran’s index analysis, after the Mann Kendall spatial analysis, are presented in Fig. 7.

## Discussion

The relationship between the occurrence of mosquito vector species and environmental and ecological conditions, which favour mosquito presence in certain locations, determines the epidemiological pattern of the various diseases whose aetiological agents are propagated by mosquitoes. Outbreaks of several vector-borne diseases are becoming more frequent in both developed and developing countries.

*Aedes aegypti* is a well-known species that is capable of colonizing multiple man-made habitats [1, 8]. The impact from its occurrence, in terms of the dispersion of virus diseases such as dengue, Zika and chikungunya, threatens public health throughout the world [17, 26]. Considering the negative impact caused by the presence of *Ae. aegypti* and the urgent need for a robust and sustainable control programme that includes monitoring the mosquito, the use of ovitraps presents advantages for entomological surveillance. By employing ovitraps to monitor the spatial–temporal distribution of *Ae. aegypti*, the costs of implementation and follow-up are low. In addition, an ovitrap is an effective instrument to monitor the presence of *Ae. aegypti* [30, 31]. The results of the analyses conducted in the present study showed that both passive ovitrap surveys and active intra- and peri-domiciliary larval collections are equally effective for monitoring *Ae. aegypti* in urban environments. However, the investment and the need for human resources in the implementation of the monitoring programme and estimation of infestation with the ovitraps represent a cost of less than 20% of that necessary for the undertaking of house-to-house surveys.

The diversity of micro-environments existing in urban areas changes the distribution of the mosaic of entomological indices, and this diversity is also an important factor in the dynamics of cases of dengue [42, 43]. In our research, we ascertained that some traps, despite the control actions taken, maintained a total number of eggs above the average during all the years of collection. In addition, the spatial correlation between the number of eggs collected in the traps and the places of storage of recyclable materials was verified because the majority of the eggs were collected at distances no greater than 270 metres from the storage locations. This information corroborates the data presented by Piovezan et al. [8], where they demonstrated that the spatial correlation between breeding sites occurs at a distance of up to 270 metres, above which value the distribution becomes random.

In the present study, the differences observed in the distribution of the eggs collected by the ovitraps within the municipal territory also occurred in the distribution of the larvae collected in the various breeding sites and in the cases of dengue. This condition clarifies that the microclimate of each suburb, consisting of its specific structural and cultural characteristics, influences the entomological and epidemiological dynamics of dengue [43].

*Aedes aegypti* is a mosquito that is highly adaptable to the ecological conditions determined by the anthropogenic environment. This species is known for its use of breeding sites produced by humans; thus, the implementation of sustainable actions, such as the collection of recyclable materials, together with the possibility of making a living from this kind of activity, can lead to the creation of environments that favour the development of the immature forms of the vector mosquito when these materials are inadequately stored. Piovezan et al. [8] observed that many materials are stored in the domestic environment for the purpose of recycling and that when stored in inadequate conditions, these materials become ideal habitats for the development of the immature stages of mosquitoes. This study demonstrated that 70% of the total number of larvae collected during the research project occurred in the kinds of breeding sites that can be used for recycling, which reinforces the need for adequate public policies for the management of waste.

Thus, it is also important to recall that in 2014, the state of São Paulo faced the greatest water-supply crisis recorded at any time in its history [44, 45]. This environmental situation led to alterations in the habits of the population affected, and among other actions, people began to store water for their daily use in containers, which altered the distribution of the larvae collected during the research project. The absence of rainfall and the population’s new habit of storing water after the water shortage crisis in 2014 meant that the reservoirs for water supply (water storage) became more important than they had been in the results found in earlier research [8]. In the three years of research, approximately 10% of all the larvae collected were found in reservoirs whose purpose was storage of water for use, which demonstrates the ability of *Ae. aegypti* to use these breeding sites as they become increasingly available.

We recognized that the presence of recyclers in the urban area was an important factor in the dynamics of the entomological data observed. The results obtained from the ovitraps and the collection of larvae clearly showed that there exists a spatial correlation between eggs/larvae and recyclers. As recycling intensifies in society, places for storing materials are increasingly scattered throughout the territory of the municipalities. To determine the influence of these locations on the dynamics of the dispersion of *Ae. aegypti* is extremely important so that actions can be directed to the places of greatest risk and that the investments made integrate training and adequate information for the target public about the management of these materials. The control of *Ae. aegypti* has little effect if its breeding sites are not eliminated to a minimum percentage of the places where the immature larvae develop [46].

It is important to understand the influence of these places, where the recyclers maintain a considerable stock of habitats for the development of mosquitoes in the neighbourhood [9]. One notable consideration is that these breeding sites often cannot be eliminated given their relation to recycling as a livelihood, and in addition, control with the application of larvicides presents slight efficacy because the ability of the product to reach the surface as necessary for treatment is often not practicable [47]. Importantly, commerce in these kinds of materials also implies the dispersal of eggs throughout the municipal territory.

The results obtained in the present study demonstrate that as other authors have reported, the environmental variations found within a particular area produce a mosaic of risk, both for identifying recipients containing *Ae. aegypti* larvae to determine the occurrence of eggs by means of ovitraps [2, 42]. Dengue is a disease that has a major economic and social impact on society, and control actions require intersectoral action by public authorities [48]. Thus, new spatial analysis tools are seen to be vitally important to helping vector control activities, permitting the search for integrated solutions for problems due to the lack of urban planning that affects collective health.

## Conclusions

Our study demonstrates that the implementation of a monitoring system with ovitraps is sensitive and agile and that its routine inclusion in dengue control actions requires few resources. Further studies should seek information that will allow the entities responsible for vector control, with a certain assurance, to possess triggers for the execution of certain actions, such as those based on the number of eggs found in a trap or the number of eggs found in a given micro-region. A clear spatial correlation exists between the places where the recyclers store their materials and the distribution of larvae and eggs in the territory. Sharing responsibility and obtaining commitments together with coordinated effort from the entire recyclables chains, involving local governments, traders, manufacturers and consumers, will be necessary for establishing a robust management and recyclables systems, and reducing the risk of *Aedes*-borne diseases. Finally, public authorities must assist implementing the correct management of recycling activities, making use of adequate practices for the handling of recyclable materials that will minimize the risks to health and strengthen social inclusion.

## Data Availability

Data supporting the conclusions of this article are included within the article. Raw data are available upon request to the first author.
